# Factors influencing the use of intermittent preventive treatment of pregnant women seeking care at primary healthcare facilities in the Bwari Area Council, Abuja, Nigeria

**DOI:** 10.4102/phcfm.v12i1.2256

**Published:** 2020-04-16

**Authors:** Grace O. Peters, Mergan Naidoo

**Affiliations:** 1 Discipline of Family Medicine, College of Health Sciences, University of Kwazulu-Natal, Durban, South Africa

**Keywords:** intermittent preventive treatment, pregnant women, malaria, antenatal care, healthcare workers, healthcare providers, Nigeria

## Abstract

**Background:**

The use of intermittent preventive treatment-sulphadoxine–pyrimethamine (IPT-SP), adapted by Nigeria, is one key preventive strategy recommended by the World Health Organization. Because antenatal clinics serve as the usual entry point for IPT-SP implementation, this study explored healthcare workers’ knowledge and practice, which are key to optimal IPT-SP coverage.

**Aim:**

This study aimed to explore the knowledge and practices of healthcare workers on the direct observation of IPT-SP amongst pregnant women attending antenatal care (ANC) in the Bwari Area Council (BWAC) of the Federal Capital Territory, Abuja, Nigeria.

**Setting:**

The study took place at five different wards of Bwari Area Council and eight Antenatal care facilities in Abuja, Federal Capital Territory, Nigeria.

**Methods:**

In-depth interviews and indirect observations were conducted among purposively selected healthcare workers in charge of the ANC of the eligible facilities. The study explored the knowledge and practices of healthcare workers on intermittent preventive treatment. Data were transcribed, translated and manually coded, and thematic analysis was conducted.

**Results:**

Healthcare workers’ knowledge of IPT-SP, mode of administration and the rationale behind the use were poor. They all agreed that IPT-SP is supposed to be administered as a directly observed therapy at the clinic, but this practice was non-existent.

**Conclusion:**

The findings indicate that factors such as adequate technical skill, accessibility and availability of logistics influence knowledge and practice of IPT service delivery. Therefore, measures should be put in place to address gaps identified by this study.

## Introduction

Malaria in pregnancy is highly prevalent in Africa and constitutes a major public health challenge in sub-Saharan Africa.^1^ Malaria affects countries worldwide − yet, 30 countries suffer 90% of the global malaria death toll.^2,3,4,5^ Just five of these – Nigeria, the Democratic Republic of Congo, Uganda, Ethiopia and Tanzania – account for 50% of global deaths and 47% of all malaria cases.^2,6,7,8^ Malaria acts as a major contributor to morbidity, including maternal anaemia, foetal loss, premature delivery and intra-uterine growth retardation, and is a risk factor for maternal and perinatal deaths.^7,9^ The financial loss because of malaria in Nigeria is estimated to be about 132 billion Naira annually in the form of treatment costs, prevention costs and loss of man-hours.^10,11^

Intermittent preventive treatment of malaria in pregnancy (MiP) using sulphadoxine–pyrimethamine (IPT-SP) is one of the three prongs of intervention designed by the World Health Organization to control MiP, especially in malaria-endemic regions where pregnant women are required to receive at least two doses of IPT-SP as early as possible in the second trimester and at every scheduled antenatal care (ANC) contact thereafter with a month’s interval till birth.^12,13^ This assumes that every pregnant woman living in a malaria-endemic area with or without symptoms of malaria has malaria parasites in her blood or placenta, with an increased susceptibility in the second and third trimesters of pregnancy.^9,14^ The IPT-SP is to be administered as a directly observed treatment during ANC visits by a qualified healthcare worker.^12^ In 2014, Nigeria adopted the updated World Health Organization (WHO) IPT-SP policy of providing IPT-SP from the early second trimester of pregnancy and then at each scheduled ANC visit until the time of delivery, provided that the doses are given at least 1 month apart.^15,16^ Because antenatal clinics serve as the usual entry point for IPT-SP implementation, the nature of service provision in the clinics as well as attendance by pregnant women is key to optimal IPT-SP coverage.

The assumption that mothers would adhere to treatment instructions if left on their own was predominantly reported among health workers.^17,18^ In 2013, the WHO stated a concern in a consensus statement that the global gains in malaria control might be lost, if the delivery of MiP interventions are still suboptimal.^19,20^ This is applicable to Nigeria, which still has a long way to go in achieving the set targets for IPT-SP.^21^ Some documented studies show low levels of IPT-SP adherence.^22^ This study explored knowledge and practice responsible for compliance with IPT-SP protocols among healthcare workers serving at primary healthcare facilities.

## Research design and methods

Abuja, the Federal Capital of Nigeria, is in the northern part of the country, at a confluence of the rivers Niger and Benue. This Federal Capital Territory has dual settlement types, both rural and urban, with the majority of the population living in the rural area. Abuja has a moderate climate, with a savanna vegetation type. The city has six area councils: Abuja Municipal Area Council (AMAC), Kuje Area Council, Gwagwalada Area Council, Kwali Area Council, Abaji Area Council and Bwari Area Council (BWAC). The population of BWAC according to the 2006 census was 229 274 (male 115 346; female 113 928).^34^

## Healthcare system

Bwari Area Council, comprising 10 wards, has two secondary health facilities, 46 primary healthcare centres and many private health facilities, chemist stores and pharmacies scattered across the council serving the needs of the populace.

### Study design

This was part of a mixed-method study (both a quantitative study using a semi-structured interviewer-guided questionnaire among 422 pregnant attendees of the eight facilities – this aspect was submitted in another journal for publication – and a qualitative study comprising non-participatory observation and in-depth interviews among ANC unit heads reported in this study) conducted in 2017. A simple random sampling (from the list of the 10 wards constituting the BWAC, every third ward was selected – the exercise was repeated to get the five wards) method was used to select five out of 10 wards that constituted BWAC, a single local government. In each of the five wards, there was at least one ANC rendering facility. In total, there were eight facilities conducting routine ANC services, and they were purposively included and sampled in the study over a period of 6 months.

On further discussion with the healthcare workers in charge of the eight facilities and the principal investigator (PI), a decision was reached to purposively interview the healthcare workers in charge (unit head) of various ANC units. This was because it was difficult to get the others involved because of shortage in staff strength and also because the ANC unit heads were thought to be information-rich participants. Among the interviewees were midwives and/or nurses, community health extension workers (CHEWs) and community health officers (CHOs).

In-depth interviews using a pretested, semi-structured tool and non-participatory observations were the main data collection methods used in this aspect of the study. The data collection among the heads of the eight selected ANC units was conducted in English by the PI (G.O.P.) and notes were taken by a research assistant. The interviews lasted 35–45 min. The interview guide was designed to extract information on their general knowledge and perception of common diseases and malaria, IPT-SP direct observation practice, barriers in the use of IPT-SP and suggestions on ways of improving IPT. However, the observation guide was used to indirectly observe protocols from point of entry, logistics availability (SP; cups; free, safe and clean potable water for direct observation treatment (DOT) at ANC; posted visual aids on malaria; and insecticide-treated nets [ITN]), interaction between patients and healthcare workers, waiting room health talk, clinical examinations, practice of DOT (swallowing of SP before healthcare workers), registers and record keeping.

Prior to the commencement of the in-depth interviews, information sheets containing the aim, objectives and processes of the study, accompanied by a consent form, were discussed. Participants who agreed to take part in the study voluntarily signed consent forms, with the option to withdraw at any stage, without any penalty. The PI with the help of a research assistant conducted eight individual in-depth interviews – face to face and telephonic – and audio-recorded the interviews in the presence of the heads of the ANC units of the eight health facilities, between January 2018 and June 2018, at a location chosen by the healthcare workers within the facility. The consent of the heads of the ANC units was sought to audio-record the discussion to maintain accuracy. Notes were taken by a research assistant, and the PI kept a reflexive journal, where impressions on the PI’s influence on the interviews were recorded.

The interviews were audio-recorded and transcribed verbatim to Microsoft Office Word 2016. To ensure validity, all transcribed interviews were cross-checked with the participants to seek clarification on issues arising from the interviews. Thematic analysis (TA) was performed to capture patterns across data. Patterns inhibiting the use of IPT guidelines were identified from the data (healthcare workers’ responses). Analysis began by repeatedly listening to the audio tapes and reading and rereading the transcribed data to ensure familiarisation. Participants’ responses were coded; codes were reviewed and grouped into categories, which were then grouped into themes. To preserve anonymity and maintain privacy, all interviewees (healthcare workers) were assigned anonymous study numbers, so that their names and health facilities cannot be traced to any participant.

### Ethical consideration

The study was approved by the university ethics committee (Reference number: BE557/17) as well as the Abuja Federal Territory Research Ethics Committee (Reference number: FHREC/2017/01/84/30-2017).

## Results

### Socio-demographic details of participants

**FIGURE 1:**
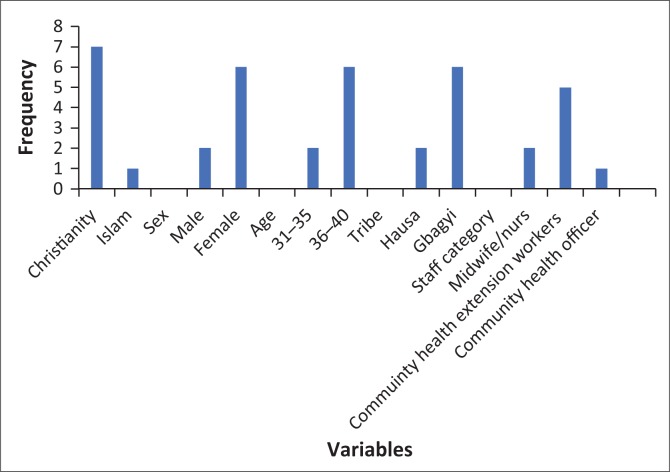
Socio-demographic characteristics of health workers.

A total of eight interviews were conducted among healthcare workers conducting and heading antenatal clinics. Socio-demographic variables are shown in Figure 1.

Six of the interviewees were females, two were midwives, five were CHEWs and one was a CHO, while seven were married and one was widowed. All participants were aged between 31 and 40 years.

## Healthcare workers’ knowledge about intermittent preventive treatment of malaria

The ability of healthcare workers conducting ANC services to apply an intervention is dependent on their understanding of the seriousness of the disease, the health system implications and the prevention and treatment interventions available. Malaria was seen as a common presenting condition in pregnant women. Healthcare workers in all facilities had a good perception of malaria; the cause, signs and symptoms; and were aware of the correct preventive measures:

‘The health challenge is malaria. Sometimes maybe abdominal pains, sometimes student maybe little or minor injury sustained by children when they are running they got wound and motorcycle accidents, road traffic accident and occasionally diarrhoea. Among children is still malaria and pregnant mothers is that malaria too that is most common.’ (Male health-worker, 30–35 years old, a CHEW)

‘Malaria is protozoa infection. Malaria is caused mostly by the bite of female infected anopheles mosquitos because there are many species of mosquitoes. Though we have different species that causes malaria. But mostly in our environment is anopheles mosquitoes, they always come up with plasmodium falciparum. When they come up with it some of the signs and symptoms some of them come up with, fever, headache, fatigue, malaise, vomiting and abdominal pains, we then do the RDT test and treat when positive, we now place them on antimalarial drugs.’ (Male health-worker, 30–35 years old, a CHEW)

‘Malaria is a disease caused by the bite of anopheles mosquitos and it is commonly found anywhere. If in a community the environment is not well kept it can easily affect children, adult and everybody else.’ (Male health-worker, 30–35 years old, a CHEW)

Most healthcare workers were aware of IPT-SP as one of the WHO interventions for MiP:

‘IPT, I think is a preventive measure for pregnant mother from having malaria. Fansidar is a drug of choice and it is giving at 16 weeks of gestation, given twice to pregnant mothers before delivery. The advice given to a pregnant mother that cannot take IPT is to stay in a clean environment, sleeps inside ITN and take proper diet for immunity to be strong to be able to fight against infection. To know when to start IPT I will palpate confirm the gestational age of the woman. I will ask when she missed her period and calculate from the month she misses her period.’ (Male health-worker, 30–35 years old, a CHEW)

‘The WHO recommended the use of Insecticide Treated Nets (ITN), Intermittent Preventive Treatment (IPT), Indoor Residual Spraying and Prompt Diagnosis and Treatment (PDT). They gave us treated bed nets and Fansidar (IPT).’ (Male health-worker, 30–35 years old, a CHEW)

### Intermittent preventive treatment-sulphadoxine–pyrimethamine timing, dosage, benefits, who cannot take and suggested alternatives

Appropriate practice and use is not only limited to their understanding of IPT but also on other factors such as timing, dosage, benefits, who cannot take IPT-SP and alternative measures. These factors were explored in the interviews and observations. Healthcare workers expressed varied knowledge about the number of times and the gestational age when IPT-SP should first be given during pregnancy. Some mentioned that the appropriate time was at 16 weeks or the second trimester, while another participant stated, ‘from 20 weeks.’ More than half mentioned twice, while two said thrice and one said twice but claimed to be aware of a new policy stating the possibility of giving IPT-SP till delivery:

‘IPT is a preventive drug giving to pregnant women to help prevent them from being infected with disease called malaria. That is drugs giving before the infection come. We give IPT (Fansidar-3 tablets at once) to pregnant women from 16–34 weeks. IPT is given two times to pregnant women before delivery. Women that are not giving IPT are advised to sleep under insecticide bed net. Women at early stage of pregnancy, that is, the first trimester (1–3 months) should not be giving IPT.’ (Male health-worker, 30–35 years old, a CHEW)

‘It is given at 16 weeks of gestation; we give the first dose at 16 weeks and give a 1-month interval appointment. We give the third dose when she complains of bitterness of mouth.’ (Male health-worker, 30–35 years old, a CHEW)

‘Three tablets at once, at least twice before delivery. Second and third trimester.’ (Male health-worker, 30–35 years old, a CHEW)

The general perception and knowledge of healthcare workers regarding the benefits of IPT-SP to a mother and her unborn child were poor. Some healthcare workers said that pregnant women in early stages of pregnancy should not take IPT-SP. Another said that any pregnant woman at any stage can be given IPT-SP. One healthcare worker mentioned that a very sick pregnant woman should not take IPT-SP. None mentioned any correct benefits of IPT:

‘You will see the foetus with good Apgar score and will not hear anything about jaundice. The early pregnant women should not be given IPT-SP, that is, the first trimester. A pregnant woman that cannot take IPT-SP, due to one reason or the other, is given health talk and advised to use net during pregnancy and delivery and clear surrounding bush.’ (Male health-worker, 30–35 years old, a CHEW)

‘Women at early stage of pregnancy, that is, the first trimester (1–3 months) should not be given IPT-SP. IPT-SP make the mother and her unborn child to be healthy and strong. A pregnant mother that cannot take IPT-SP is to stay in a clean environment, sleep inside ITN and take proper diet. Women that are not given IPT-SP are advised to sleep under insecticide bed net.’ (Male health-worker, 30–35 years old, a CHEW)

‘Women that come for ANC and take IPT-SP don’t come up with malaria. There is no pregnant woman that should not be given IPT-SP based on any ailment. For pregnant women that cannot take IPT-SP, they should sleep under net.’ (Male health-worker, 30–35 years old, a CHEW)

## Healthcare workers’ practice

Healthcare workers agreed that the stipulated recommended mode of administration is by direct observation. However, all facilities/wards gave IPT-SP along with other routine drugs as take-home drugs:

‘We do include the IPT-SP in their routine drugs. Initially we do give them to take the drugs at the facility once a month, some do complain of not eating before coming to the facility.’ (Male health-worker, 30–35 years old, a CHEW)

None of the facilities visited actively promoted or practised DOT. One healthcare worker claimed that DOT of IPT was formally observed, but this practice was stopped based on complaints from pregnant women. Respondents in all the facilities claimed that the main reason for not carrying out DOT was because of pregnant women complaining of not eating before coming to the facility and therefore they were not ready to take the drug on an empty stomach:

‘Initially we do give them to take the drugs at the facility once a month, but some do complain of not eating before coming to the facility. So, we do give the drugs to them and advise them to take the drug at home. We do include the IPT drugs in their routine drugs. We do encourage them on the importance of taking their IPT drugs at home.’ (Male health-worker, 30–35 years old, a CHEW)

The knowledge of the mode of operation, importance, benefits and mode of IPT-SP dissemination was generally poor in all eight facilities across the five wards studied.

## Intermittent preventive treatment logistics and access

Successful implementation of malaria prevention in pregnancy depends on the availability of IPT-SP, clean potable water, cups, ITNs, registers, protocols, training manuals and visual aids. This, in turn, depends on procurement (Area Council Central Drug Store, open market, prescription) and the management of the logistics. This assumption was confirmed in all facilities across the wards.

### Availability

There was a consensus among all healthcare workers that IPT-SP drugs were usually available; however, there also used to be occasional drug stock shortages. From observation, only one facility had a visible water point with cups for pregnant women. In terms of ITN, all the facilities claimed not to have received any in the previous few months. None of the facilities had training manuals or guidelines on malaria at the time of study:

‘So, we have our drugs. They normally supply us with drugs. Drug is available most times because of the Drug Revolving Fund (DRF) and the supply from WHO.’ (Male health-worker, 30–35 years old, a CHEW)

‘We do have, because this drug is free, IPT-SP. We are able to get our supply most time before next clinic.’ (Male health-worker, 30–35 years old, a CHEW)

The early arrival of the PI and field workers at each facility prior to the start of clinic activities allowed for the observation of the general interaction of the healthcare workers and their clients. Healthcare worker–client interaction was generally good in all facilities of the study. Although health talks were conducted at all facilities, MiP was not a major discourse in six facilities, with few mentioning IPT-SP. Although malaria was mentioned, other issues of diet, environmental sanitation and birth preparedness took most of the time. Time constraints and the large numbers of pregnant mothers in attendance were factored into the time dedicated to health talk. Five facilities had SP at the time of visit. Seven facilities did not have clean water visibly available. None of the healthcare facilities practised DOT. No health education programme plan was observed. No facility had IPT-SP National Protocols, training manuals and ITNs for distribution. Some pregnant women paid for some elements of their healthcare. Other findings based on non-participatory observation are shown in Table 1.

**TABLE 1:** Non-participatory observations.

Observable variable	Yes	No
**Availability of logistics and observed practice of healthcare workers**		
Health education programme drawn for the quarter includes MiP	0	8
Health education programme drawn for the quarter includes IPT	0	8
Health talk given at ANC on day of visit	8	0
Health talk given that day included malaria in pregnancy	5	3
Health talk given that day included IPT	1	7
Presence of posters of IPTp/MiP on the wall	0	8
Presence of ANC report book for daily summaries	8	0
Presence of ANC Monthly Data Returns Form	8	0
SP available at ANC	5	3
Practice of DOT observed	0	8
SP given is recorded in ANC report book for daily summaries	5	3
SP given is recorded in ANC book of clients	4	4
Presence of Adverse Event Forms for SP	0	8
Presence of free, clean, safe water for DOT	1	7
Presence of safe, clean water for sale for DOT	0	8
Availability of IPT National Protocol	0	8
Availability of IPT training manual	0	8
Presence of ITNs for distribution to clients	0	8

ANC, antenatal care; MiP, malaria in pregnancy; ITN, insecticide-treated nets; DOT, direct observation treatment; SP, sulphadoxine–pyrimethamine; IPT, intermittent preventive treatment.

### Accessibility and procurement

The main source of IPT-SP procurement in all facilities was the BWAC drug store. All claimed that the IPT-SP was readily accessible most of the time. However, because of occasional drug stock shortages, all healthcare workers across facilities sometimes procured drugs from the private sector and operated a drug revolving fund (DRF):

‘We get our supply from the area council drug store. In case area council doesn’t have, we buy from open market. We go to another facility, because we have our sister clinic here.’ (Male health-worker, 30–35 years old, a CHEW)

Some of the facilities claimed to get supplies of IPT-SP from other sources apart from the area council and the private sector. In spite of occasional stock shortages, nearly all the participants reported that they infrequently write prescriptions for pregnant woman attending their facilities:

‘In case of stock out we do buy from the open market because we cannot keep them waiting IPT. Either we issue or we prescribe for them to buy.’ (Male health-worker, 30–35 years old, a CHEW)

## Barriers experienced by the healthcare workers

The healthcare workers expressed various barriers experienced in the course of discharging their duties pertaining to IPT-SP. Healthcare workers across all facilities expressed concerns about the lack of ongoing training in terms of updating their knowledge. Their concerns ranged from a lack of exposure to recent developments in IPT administration, to erratic post-institutional training after school. They all unanimously agreed that there is a need for ongoing training for better and more efficient performances. None mentioned the lack of clean water and cups as a barrier, while nearly all facilities surveyed lacked water points and cups:

‘Now when this global training, there is a lot of new, new thing that are coming up that we suppose to know and which we don’t know.’ (Male health-worker, 30–35 years old, a CHEW)

Healthcare workers claimed to enjoy the good will of clients using the facilities. However, these attitudes sometimes changed especially when drugs were not available. Antenatal care services are free at primary healthcare level and the introduction of payment at a subsidised rate through the DRF to cater for the occasional stock shortages placed pregnant women under financial strain. As a result of this, some claimed to have SP when they did not.

‘Clients have to buy the drugs at a subsidised rate, but when it is supplied by the area council it is free.’ (Male health-worker, 30–35 years old, a CHEW)

## Suggestion for improvement

In general, all healthcare workers suggested that there should be an improvement in the logistics in terms of availability (especially IPT-SP, ITN), accessibility, including training and re-training (capacity building) for all healthcare workers and facilities. Most of the healthcare workers claimed that these might enhance the practice of DOT implementation of IPT-SP in all facilities:

‘If the government will make IPT drugs to be adequately available in all facility and also make ITN available. This will enable all pregnant women to be able to get their drugs irrespective of their financial status.’ (Male health-worker, 30–35 years old, a CHEW)

## Discussion

The aim of this study was to examine the knowledge of healthcare workers and the practice of DOT of IPT (IPT-SP) amongst pregnant women attending ANC in BWAC of the Federal Capital Territory, Abuja. This study revealed several factors, including knowledge, non-availability of supplies and accessibility of logistics, that inhibited the practice and use of IPT-SP guidelines.

Healthcare workers’ knowledge and understanding with respect to IPT guidelines were generally poor. Although they were within the correct limits on the gestational age when taking the first dose of IPT-SP and the dosage, they all demonstrated poor knowledge of the current updated IPT guidelines. Nigeria adopted the updated guidelines that stipulate that IPT-SP should be used until delivery.^12^ One healthcare worker, however, expressed awareness of the new guidelines but was not certain that the policy was being implemented within the healthcare system. This finding was supported by a similar study in Malawi that reported that a poor level of knowledge of the guidelines for IPT-SP delivery amongst healthcare workers negatively affected IPT-SP delivery.^23^ In another study by Honkasalo and Launiala,^24^ healthcare workers offered all women IPT-SP (including first trimester clients) during their first clinic visit.

Their understanding of the benefits of IPT-SP for a pregnant woman and her unborn child were poor. This confirmed the findings of a study by Onoka et al.^25^ in 2012, which confirmed that poor practice or non-use of IPT-SP guidelines could be the result of poor knowledge. Another study by Yoder and others^23^ in Malawi, which revealed that a poor level of knowledge of the guidelines for IPT-SP delivery amongst healthcare workers negatively affected IPT-SP delivery, further corroborated the findings of the study. In this study, in spite of good attendance of antenatal clinics and pregnant women’s trust and reliance on healthcare workers, the use of IPT-SP might not be optimal because of the poor knowledge of the healthcare workers on IPT-SP guidelines and benefits. Studies have shown that ANCs are considered an important entry point to target the pregnant women.^22^ Around 60% – 70% of women attend ANC clinics at least once during pregnancy in Nigeria.^22,26^

This study also confirmed a previous study by Olorounfemi^27^ and others who reported that a gap between coverage and targets indicated missed opportunities. In another study conducted in Enugu, Nigeria, the level of access to IPT-SP for MiP was still low in spite of relatively high ANC coverage in the study area.^28^ A study in Nigeria by Fawole and Onyeaso^29^ reported a low level of knowledge of IPT guidelines amongst a sample of all categories of healthcare providers, which was consistent with our study. The use of DOT to deliver IPT-SP needs to be looked at from a systems point of view, and innovative methods, such as the provision of an incentive and the training of nursing assistants to ensure that IPT-SP^28^ is taken, may help strengthen the healthcare system and improve malaria outcomes.

Direct observation administration practice of IPT-SP was not used by any of the healthcare workers in this study. This finding was not surprising because their perception and understanding of the guidelines of IPT-SP was poor. Healthcare workers explained that IPT-SP is given along with other routine drugs to the pregnant women. Poor healthcare worker practices have also been identified among others as operational challenges in delivering IPT; this was revealed in a study by Hill and Kazembe^18^ on the review of progress and operational challenges in reaching the Abuja target for IPT of MiP in African women. Similarly, a study conducted in Korogwe district of northeast Tanzania, by Mubyazi^23^ and others, found that only a quarter of pregnant women took SP under observation by a healthcare worker; this further corroborated the current study’s findings on poor compliance to direct observation by healthcare workers.

It was clearly shown in their responses that they were not aware that IPT-SP is safe and could be taken on an empty stomach. This study was in line with another study conducted in Malawi, where some healthcare workers were of the opinion that SP should not be taken on an empty stomach, which reduced the delivery of SP under direct observation.^23^ However, the guidelines on IPT use and some studies refuted the claim that SP cannot be taken on an empty stomach.^12,30,31^ This summation by the healthcare workers further supported the need for further training to strengthen their knowledge as requested by all participants.

In this study, healthcare workers expressed experiencing occasional stock shortages of SP for IPT-SP in their facilities. A similar study by Tarimo^32^ reported that 40% of women interviewed in Tanzania had not received SP because of its unavailability, suggesting that stock shortage is a major barrier to malaria prevention. Additional expense in the form of payment for SP, through the DRF schemes, was a barrier to the use of IPT. This was confirmed by many respondents (healthcare workers) who noted that some pregnant women abscond from attending future clinics, while others claim to have some SP left to avoid purchasing another set. These inequalities may, to some extent, reflect the determinants of women’s access to ANCs, where user fees are routinely applied to registration, consultations, laboratory tests and drugs, as identified in a review of factors affecting utilisation of ANC in developing countries.^33^

**FIGURE 2:**
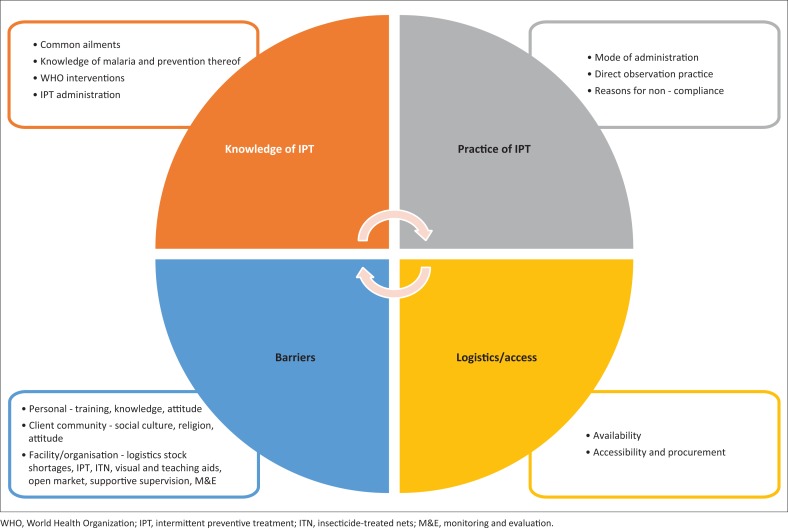
Factors determining the use or non-use of intermittent preventive treatment-sulphadoxine–pyrimethamine in Bwari Area Council.

Findings from the above study expressed issues affecting healthcare workers’ practice of direct observation, use of IPT-SP *vis-à-vis* the mode of administration by direct observation and reasons for noncompliance as: their general knowledge of common ailments, malaria and its prevention, WHO interventions was poor. Access and logistics (availability, accessibility and procurement) of SP; potable water and cups; and various barriers at personal, client and facility levels hamper the useful practice of IPT-SP as reported by healthcare workers interviewed in the course of the study. Findings are summarised diagrammatically in Figure 2.

### Strengths and limitations

One of the limitations of the study was that, firstly, it took place in only one Area Council of Abuja, the Federal Capital Territory, with a total of just six area councils. Secondly, the interview took place within the premises of the facilities. A number of techniques were used to improve trustworthiness and achieve a comprehensive understanding of the findings: data from a variety of participants and different sources (interviews and observations) were triangulated, as well as verification of information in records and health registers.

## Conclusion

This study revealed that healthcare workers were not implementing IPT-SP guidelines appropriately by direct observation. This may be connected to their poor knowledge, inadequate technical skills, absence of IPT-SP guidelines and occasional stock shortages of IPT-SP. For any positive change to occur, all hands must be on deck to address the unacceptably high statistics of malaria incidence and maternal mortality figures in the country. These findings highlight issues to be addressed to achieve a positive change. These include (1) poor adherence to the DOT scheme, (2) low levels of awareness and understanding on recently adopted WHO guidelines on intermittent preventive treatment (IPT), (3) periodic stock shortages of IPT-SP, (4) inadequate training and re-training, (5) absence of water points and cups and (6) inadequate staff strength.

Interventions to address the gaps and deficiencies should include building capacity of health workers through training and continuing education, to improve their knowledge about malaria during pregnancy and in particular the IPT-SP strategy and DOT scheme. Simplified versions of IPT guidelines should be produced and made available to all primary healthcare centres. Health promotion packages in the form of visual aids and posters explaining the benefits and safety of IPT-SP should be developed and made available to all primary healthcare centres (PHCs). There should be a planned schedule for the procurement of SP to avoid shortages that usually lead to payment for drugs, although at a subsidised rate, through the drug-revolving scheme in all healthcare facilities; it may be noted that provision of SP is supposed to be a free service, provided for all pregnant women attending primary healthcare facilities. Efforts should also be made to provide portable water at ANCs to be used by the clients to take SP under DOT because DOT is necessary to measure the coverage of IPT. Performance-based rewards can be introduced to motivate compliance to direct observation in dispensing SP in primary healthcare facilities. In conclusion, supportive supervision and regular monitoring of all PHCs should be given priority.
